# Development and Validation of a Rapid and Simple UHPLC–MS/MS Method for the Determination of Colchicine in Human Plasma

**DOI:** 10.1002/bmc.70222

**Published:** 2025-09-23

**Authors:** Nela Žideková, Kristián Pršo, Marek Pršo, Miloš Jeseňák, Oldřich Farsa, Martin Kertys

**Affiliations:** ^1^ Biomedical Center Martin, Jessenius Faculty of Medicine in Martin Comenius University Bratislava Martin Slovak Republic; ^2^ Department of Research and Development Saneca Pharmaceuticals Hlohovec Slovak Republic; ^3^ Department of Pediatrics and Adolescent Medicine, Jessenius Faculty of Medicine in Martin Comenius University Bratislava, University Hospital Martin Martin Slovak Republic; ^4^ Institute of Clinical Immunology and Medical Genetics, Jessenius Faculty of Medicine in Martin Comenius University Bratislava, University Hospital Martin Martin Slovak Republic; ^5^ Department of Chemical Drugs, Faculty of Pharmacy Masaryk University Brno Czech Republic; ^6^ Department of Pharmacology, Jessenius Faculty of Medicine in Martin Comenius University Bratislava Martin Slovak Republic

**Keywords:** colchicine, familial Mediterranean fever, liquid chromatography–tandem mass spectrometry, phospholipid removal, therapeutic drug monitoring

## Abstract

Colchicine is a naturally occurring alkaloid primarily derived from plants of the Colchicum genus, which is used to treat gout and serve as a frontline therapy for various inflammatory conditions, including familial Mediterranean fever. Although it is not recommended for routine therapeutic drug monitoring, there are situations where it may be beneficial, such as in dose adjustments. The present study introduces an LC–MS/MS method for quantifying colchicine in human plasma. A one‐step extraction procedure employing an Ostro plate was applied, and the extracts were analyzed using gradient elution followed by detection on a mass spectrometer in multiple reaction monitoring mode. Our method offers several advantages, including a low sample volume and a run time of only 3 min. It demonstrates sufficient linearity to quantify low and high concentrations of colchicine in human plasma samples. The method was successfully validated in accordance with the ICH guideline M10 on bioanalytical method validation, covering selectivity, linearity, limit of quantification, accuracy, precision, dilution integrity, carry‐over effect, matrix effects, extraction recovery, and stability over a concentration range of 0.05–100 ng/mL. The fully developed and validated method was applied to determine colchicine in plasma samples from patients diagnosed with familial Mediterranean fever.

## 1 Introduction

**FIGURE 1:**
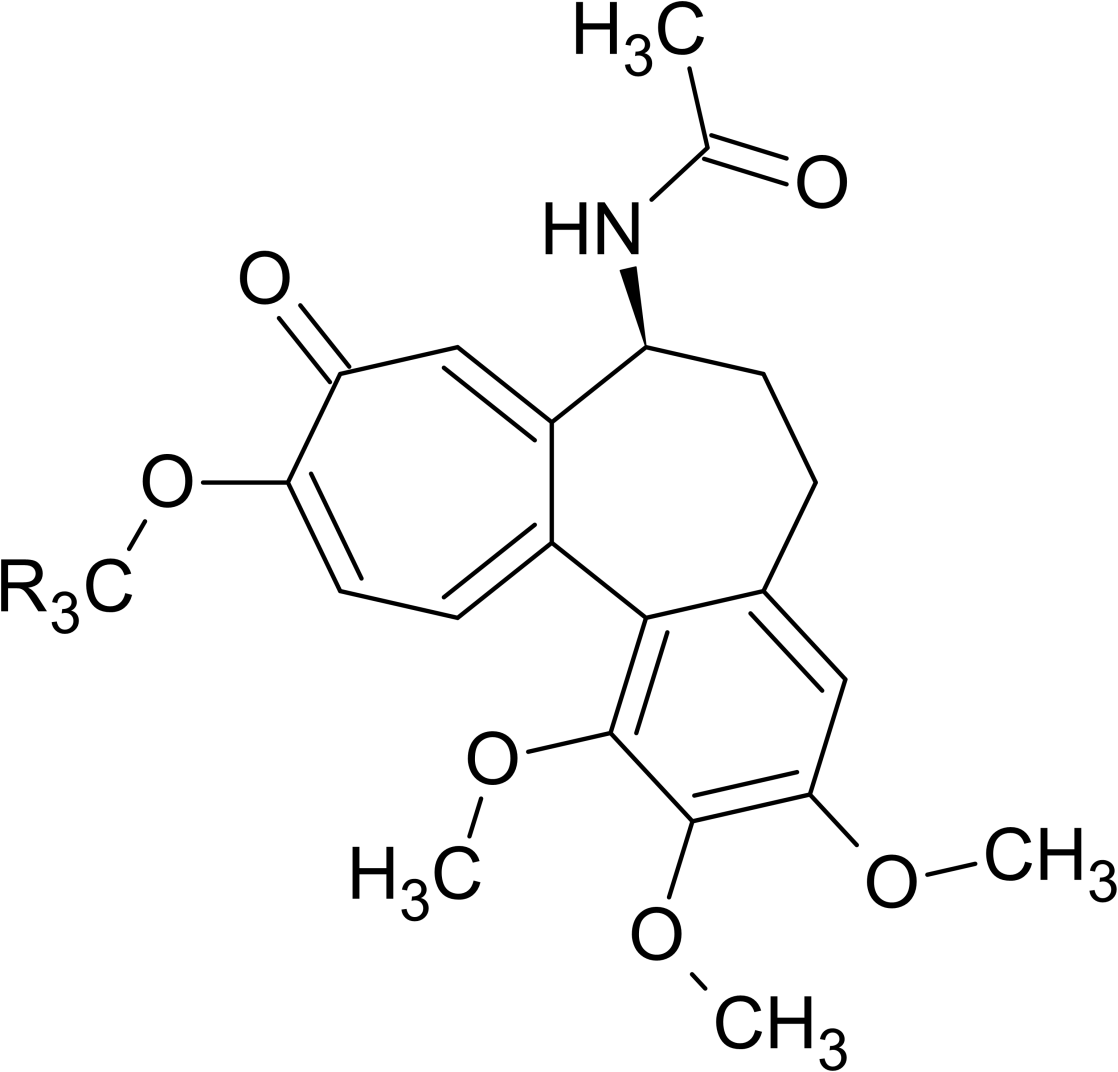
Chemical structures of colchicine (R=H) and colchicine‐d_3_ (R=^2^H).

Colchicine (COL; N*‐[(7S)‐1,2,3,10‐tetramethoxy‐9‐oxo‐6,7‐dihydro‐5H‐benzo[a]heptalen‐7‐yl]acetamide*) is a naturally occurring alkaloid primarily derived from plants of the Colchicum genus, particularly *Colchicum autumnale* (autumn crocus or meadow saffron) (Slobodnick et al. 2015). Historically used for the treatment of gout, COL remains a frontline therapy for various inflammatory conditions, including familial Mediterranean fever (FMF) and pericarditis (Bustaffa et al. 2025; Leung et al. 2015). Structurally (Figure 1), it is a tricyclic compound with notable physicochemical properties, including low aqueous solubility and high lipophilicity, which influence its pharmacokinetic profile (Finkelstein et al. 2010). The pharmacological action of COL is mainly attributed to its binding to β‐tubulin, thereby inhibiting microtubule polymerization and affecting a range of cellular processes, including mitosis, neutrophil motility, and inflammatory cytokines secretion (Jordan and Wilson 2004). This mechanism has also spurred interest in its use in oncology and cytogenetics (Kumar et al. 2016). However, colchicine's narrow therapeutic index demands careful dose management to mitigate potential toxicity (Wu and Liu 2022).

Peak plasma levels are reached 1–3 h after oral administration. Due to its extensive distribution in leukocytes, COL may be detectable in these cells up to 10 days after the intravenously administered dose. Additionally, COL has a high potential for drug–drug interactions as it is a substrate for particular cytochrome enzymes and P‐glycoprotein (Stamp and Barclay 2014). However, COL is not generally regarded as a candidate for therapeutic drug monitoring (TDM). On the other hand, there may be specific scenarios in which TDM could be considered, such as in patients with certain renal or hepatic impairments, or when there are concerns regarding toxicity or non‐response to treatment. Currently, no studies have specifically aimed to determine the plasma level range associated with the therapeutic zone of COL. However, when comparing plasma level data from patients to the clinical signs noted by prescribing clinicians, it appears that plasma levels between 0.5 and 3 ng/mL are linked to therapeutic effects (Niel and Scherrmann 2006).

Currently, several liquid chromatography‐based methods for COL quantification in various biological matrices, such as blood plasma and urine, in combination with ultraviolet (Pietsch et al. 2008; Samanidou et al. 2006) or mass spectrometry detection (Bi et al. 2022; Bourgogne et al. 2013; Canbolat 2022; Fabresse et al. 2017; Gabani et al. 2020; Jiang et al. 2007; Qian et al. 2023; Shah et al. 2014), have been published. However, not all methods are suitable for clinical laboratories due to disadvantages such as longer run times, a relatively large sample volume, and time‐consuming or costly sample pre‐treatment that diminishes sample throughput. Among the methods above, the lowest limit of quantification (LOQ) was 0.01 ng/mL, as reported by Shah et al. (2014); however, the majority of methods presented an LOQ above 0.2 ng/mL. A similar situation arises in the case of the sample necessary, where the method published by Gabani et al. (2020) requires 50 μL of plasma sample. In contrast, the other methods work with 100 μL of sample or more. Table S1 (Supplementary file) summarizes the overview of these methods. Considering the low levels of COL in blood and the potentially wide concentration range (especially in cases of overdosing), the combination of liquid chromatography with tandem mass spectrometry detection appears to be an ideal analytical technique for quantification, owing to its powerful and sensitive nature in performing TDM on multiple samples (Kilianova et al. 2024; Maekawa and Mano 2022; Maráková et al. 2013).

In this paper, the aim was to develop and validate a sensitive and simple liquid chromatography–tandem mass spectrometry (LC–MS/MS) method for quantifying COL in human plasma. A one‐step preparation procedure employing a 96‐well plate for protein precipitation and phospholipid removal was utilized for sample processing. Furthermore, the incorporation of stable isotope‐labeled COL as an internal standard (IS) improved the precision and accuracy of the measurement. The method was subsequently validated in accordance with the International Council for Harmonization of Technical Requirements for Pharmaceuticals for Human Use (ICH) guideline M10 on bioanalytical method validation and the European Medicines Agency (EMA) Guideline for bioanalytical methods validation, respectively. Finally, the method was applied to samples from patients treated with COL who were diagnosed with FMF.

## 2 Materials and Methods

### 2.1 Chemicals and Reagents

The reference standard of COL (HPLC purity: 99.2%; batch: SVI‐ALS‐15‐069) was purchased from Alsachim (Illkirch‐Graffenstaden, France). The reference standard of colchicine‐d_3_ (COL‐d_3_) in acetonitrile (100 μg/mL; isotopic purity, 99.7%; batch number: FN06092103) was purchased from Cerilliant (Round Rock, TX, USA). Acetonitrile, methanol, water, ammonium formate, and formic acid were of LC–MS grade and obtained from Honeywell Riedel‐de Haën (Seelze, Germany). The analyte‐free human plasma (hereafter referred to as blank plasma), with potassium ethylenediaminetetraacetic acid as the anticoagulant, was obtained from healthy, untreated volunteers (aged 25–30 years, both men and women).

### 2.2 Instrumentation and LC–MS/MS Conditions

The XEVO TQ‐S triple quadrupole mass spectrometer system (Waters; Prague, Czech Republic) was employed and operated in ESI mode to detect COL. Detection was achieved using positive electrospray ionization, with the ion source parameters configured as follows: capillary voltage, 1.0 kV; source offset, 50 V; and ion source temperature, 150°C. Nitrogen served as the desolvation gas at a flow rate of 1000 L/h and a temperature of 600°C. The mass spectrometer was configured for a multiple reaction monitoring (MRM) experiment at unit mass resolution (0.75 Da). Collision‐induced fragmentation was conducted using argon at a flow rate of 0.15 mL/min. Collision energies and cone voltages were optimized for COL and COL‐d_3_ standard (Table S2, Supplementary file). Data were acquired using MassLynx software, version 4.2, and were calibrated and quantified by TargetLynx software (both from Waters; Prague, Czech Republic).

The ACQUITY UPLC system (Waters; Prague, Czech Republic) consists of a binary gradient pump, an autosampler featuring a flow‐through needle design, and a column thermostat, all of which are coupled to a mass spectrometer. Chromatographic separation was achieved on an ACQUITY UPLC BEH C18 (50 × 2.1 mm, 1.7 μm) column, fitted with a 0.2 μm in‐line filter (column and filter were purchased from Waters; Prague, Czech Republic). The column temperature was set at 40°C, while the autosampler temperature was maintained at 8°C. The separation of analytes was accomplished using a gradient elution program over 3 min using 10 mM ammonium formate in water (mobile phase A) and acetonitrile (mobile phase B) at an initial flow rate of 0.4 mL/min (Table S3, Supplementary file). The injection volume was four microliters.

### 2.3 Preparation of Calibration Standards and Quality Control Samples

A stock solution for COL was prepared from its reference standards in pure acetonitrile (approx. concentration 3 mg/mL). Afterwards, a series of eight working solutions was prepared by diluting the appropriate volume of stock solution with a 50:50 (*v/v*) methanol/water mixture to achieve a concentration range of 1–2000 ng/mL. Moreover, using an independently prepared stock solution, four working solutions were prepared for quality control (QC) samples. The working solution for IS was prepared using the respective reference standard in methanol (final concentration 77 ng/mL). All the solutions were stored at −80°C.

The calibration standards and QC samples were created by spiking 950 μL of blank plasma with 50 μL of the corresponding working solutions. In this manner, eight calibration standards were produced at final concentrations of 0.05, 0.10, 0.50, 1.0, 5.0, 10, 50, and 100 ng/mL. QC samples were prepared similarly at four concentration levels (LOQ, low, medium, and high): 0.05, 0.15, 40, and 80 ng/mL. The calibration and QC samples were freshly prepared immediately prior to each analysis.

### 2.4 Sample Processing

Plasma samples, calibration standards, and QC samples were processed as follows: a 50‐μL aliquot of plasma samples and 20 μL of IS working solution were pipetted into an Ostro 96‐well plate (Waters; Prague, Czech Republic), which was positioned on a 1‐mL collection plate. First, the mixture was homogenized by vortex mixing for 1 min at 550 rpm. Subsequently, 150 μL of ice‐cold 1% formic acid in acetonitrile was rapidly pipetted into the wells and mixed by aspirating 10 times using an eight‐channel pipette. After mixing, the plate was placed onto an Otto SPEcialist Positive Pressure Manifold (Waters; Prague, Czech Republic), and nitrogen pressure at 40 psi was applied for 2 min. Afterwards, the eluate was evaporated to dryness at 60°C using a vacuum concentrator (Concentrator Plus, Eppendorf; Hamburg, Germany) over 20 min. The dry residues were dissolved in 50 μL of 15% methanol in water, shaken at 750 rpm for 5 min, and then analyzed.

### 2.5 Method Validation

The method was validated in accordance with the recommendations of the ICH guideline M10 on bioanalytical method validation (ICH M10 on Bioanalytical Method Validation—Scientific Guideline|European Medicines Agency 2022). The validation criteria included selectivity, linearity of the calibration curve, LOQ, accuracy, precision, dilution integrity, carry‐over effect, extraction recovery, and stability under various conditions. The matrix effects have been evaluated in accordance with the EMA guideline for bioanalytical methods validation (European Medicines Agency, Guideline on bioanalytical method validation 2012).

The method's selectivity was evaluated by monitoring the quantification and qualification MRM transitions of COL and COL‐d_3_ in drug‐free human plasma from six different sources, and subsequently compared to the same blank plasma samples spiked with analytes at LOQ. Selectivity was guaranteed if the interference due to endogenous substances was < 20% and < 5% of the mean peak response of the analyte and IS, respectively.

Eight calibration points were employed to construct the calibration curves within the concentration range of 0.05–100 ng/mL across three independent days. Calibration curves were established by plotting peak area ratios (analyte/internal standard) against nominal plasma concentrations. A linear weighted least‐squares analysis was conducted, with a weighting factor of 1/x^2^ selected. A coefficient of determination (*r* ^*2*^) greater than 0.98 was anticipated. The lowest calibration point was deemed LOQ if the signal‐to‐noise (S/N) ratio exceeded 10. Moreover, the upper limit of quantification (ULOQ) was regarded as the highest calibration point.

The intra‐day precision and accuracy were evaluated in six replicates at four QC levels (LOQ, low, medium, and high concentration levels) within a single day on the same analytical run. In contrast, inter‐day precision and accuracy were assessed by analyzing QC samples over three consecutive days. The coefficient of variation (CV, %) served as the parameter for estimating precision, while accuracy was represented as a percentage of the nominal concentration (%). Precision and accuracy should fall within 20% at LOQ and 15% for the low, medium, and high QC levels.

To verify the integrity of the dilution, blank plasma samples were spiked in six replicates at a concentration 10 times the ULOQ (1000 ng/mL). Subsequently, these highly concentrated plasma samples were diluted tenfold with blank plasma before the extraction procedure. Accuracy and precision within ±15% were established as acceptance criteria.

The carryover effect was determined by comparing extracts of blank plasma injected immediately after the highest calibration standard was injected in technical triplicate. The extract of blank plasma should show no significant response at the retention times for COL and IS.

The matrix effects were assessed by comparing the peak areas obtained from six individual drug‐free plasma samples. The extracts of plasma samples were spiked with standard solutions of COL at two QC levels (low and high), along with an IS solution, and compared to a pure reference standard solution in water at equivalent concentrations. The normalized IS matrix factors were calculated by dividing the analyte's matrix factor by the corresponding IS matrix factor. The CV of the normalized IS matrix factors should not exceed 15%. The extraction recovery from plasma for COL was calculated by comparing the peak areas of QC samples at two levels (low and high) with those of the blank sample extracts spiked with analytes at the same concentration (post‐extraction spiked plasma samples). The extraction recovery was investigated in plasma samples obtained from six individual sources.

The stability of COL in human plasma was confirmed by analyzing six replicates of spiked plasma samples at two concentration levels (low and high QC) under various storage conditions. Short‐term stability was assessed after the samples were exposed to laboratory temperature for 16 h. Freeze–thaw stability was evaluated after three freeze–thaw cycles, spanning a temperature range from −20°C to laboratory temperature. Post‐processing stability was determined after 24 h of storage in the autosampler at 8°C. Finally, long‐term stability was established following 2 months of storage of spiked samples in a freezer at −20°C. The accuracies were calculated as the difference between the initial calculated concentration (c_t0_) and the concentration at a specific time point (c_tx_), using the following formula: c_tx_/c_t0_ × 100%.

### 2.6 Method Application

The validated method was used to quantify COL in samples collected from patients diagnosed with FMF. The Ethics Committee approved the study protocol of the Jessenius Faculty of Medicine in Martin, Comenius University in Bratislava (Slovak Republic)—approval protocol number: EK 20/2019. Patients enrolled in the study had been taking COL under approved indications for at least 2 weeks at a stable dose to ensure steady‐state levels. Eight patients (seven females, one male) with an average age of 44.5 ± 12.2 years were included in the study.

Blood samples were drawn within 5 h following drug administration. Blood was collected in a tube containing potassium ethylenediaminetetraacetic acid as an anticoagulant, centrifuged at 2500 ×*g* for 10 min at room temperature, and stored at −20°C until analysis.

## 3 Results and Discussion

### 3.1 Method Development and Optimization

To date, several analytical methods have been published to determine COL in human plasma samples; however, not all are suitable for real clinical conditions. The limitations of these methods include the requirement for larger sample volumes (Bi et al. 2022; Canbolat 2022; Pietsch et al. 2008), lengthy analysis times (Bourgogne et al. 2013; Canbolat 2022; Pietsch et al. 2008; Qian et al. 2023), and laborious sample preparation procedures (Qian et al. 2023; Shah et al. 2014). Therefore, in the present study, we developed an LC–MS/MS method for the quantification of COL, which requires only a 50‐μL sample volume, facilitating easy and high‐throughput sample processing, with an analysis time of 3 min per sample.

**FIGURE 2:**
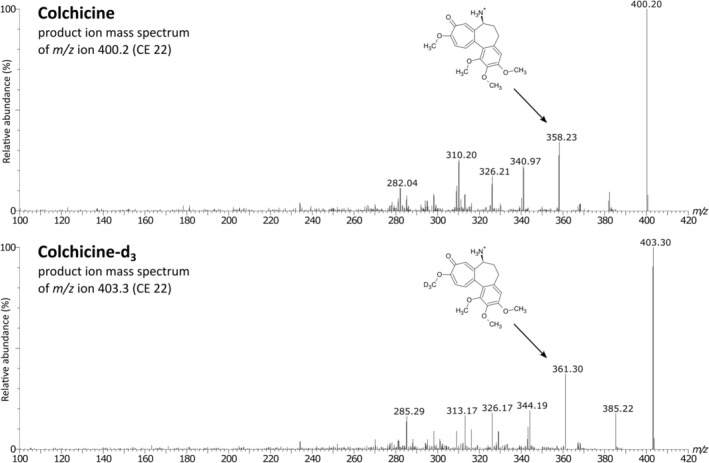
The MS/MS product ion mass spectra of colchicine and colchicine‐d_3_ with proposed fragmentation patterns.

Initially, method development started with optimizing the detector parameters of the mass spectrometer. The ionization and fragmentation conditions for COL and IS were achieved by continuously infusing the analytes dissolved in methanol/water (50:50, v/v) using the mass spectrometer's internal fluidic pump. The positive ESI mode was selected, with the full scan mode (Q1 scan) allowing the observation of the protonated ions [M + H]^+^ for COL and COL‐d_3_ at *m/z* 400.2 and *m/z* 403.3, respectively. The optimal detector response was recorded at 1.0 kV for capillary voltage and 600°C for desolvation temperature. Subsequently, collision‐induced fragmentation was used to produce fragments, with the most intense observed at *m/z* 358.2, *m/z* 326.2, and *m/z* 310.2 for COL, while the product ions of COL‐d_3_ were *m/z* 361.3 and *m/z* 313.2. The MS/MS parameters, including collision energy and cone voltage potential, were optimized to maximize signal intensity for all transitions (Table S2, Supplementary File). The product ion spectra, along with proposed fragmentation patterns, are illustrated in Figure 2.

**FIGURE 3:**
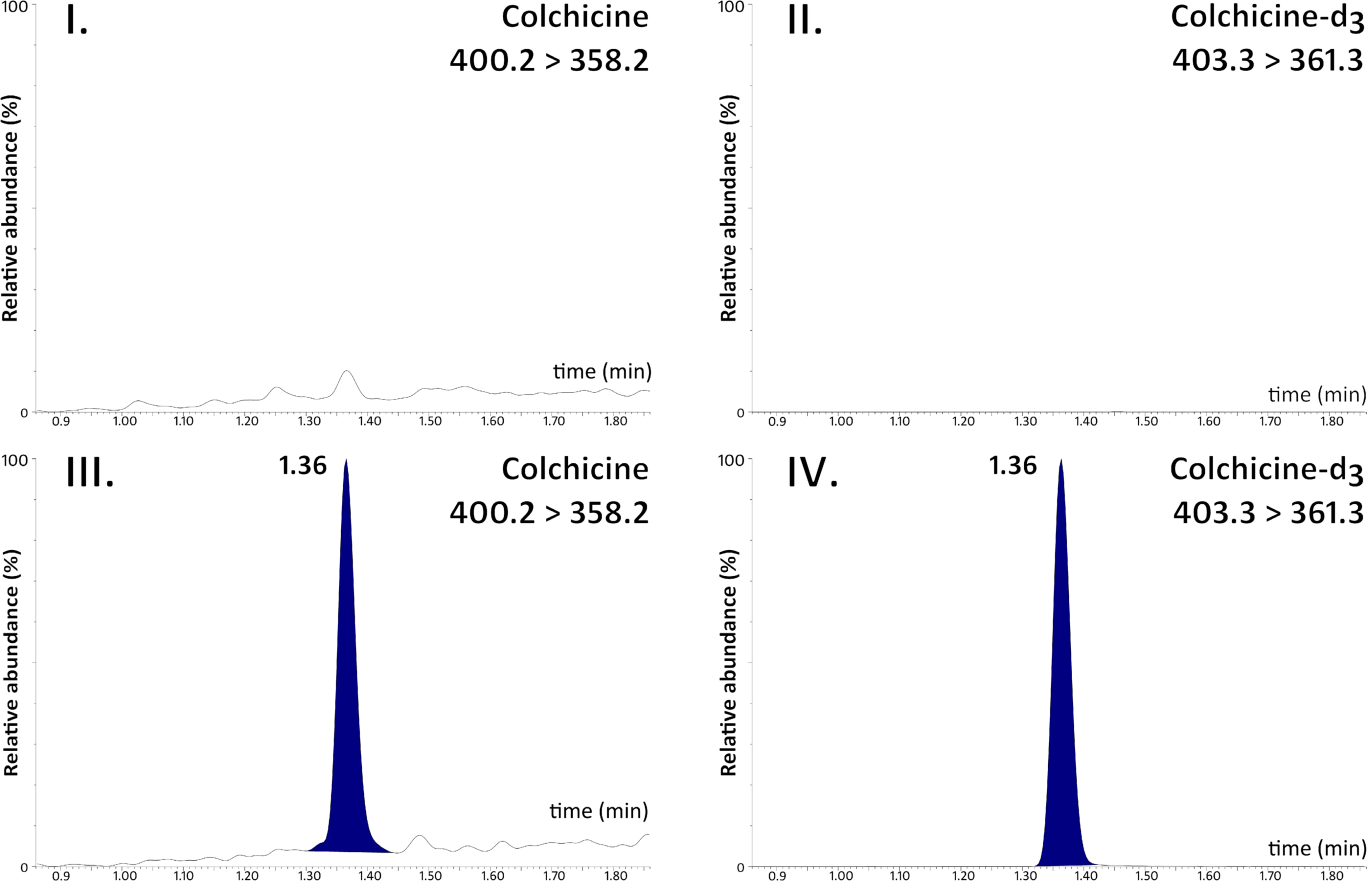
Representative chromatograms of blank plasma (I. MRM of colchicine, II. MRM of colchicine‐d_3_) and blank plasma spiked with a standard solution at LOQ (III. MRM of colchicine, IV. MRM of colchicine‐d_3_). The retention time for both COL and COL‐d_3_ is 1.36 min. The *x*‐axis represents retention time (min), and the *y*‐axis represents the relative abundance (%).

The chromatographic conditions were then optimized to effectively separate COL from endogenous interferences, achieve adequate retention time, and obtain a symmetrical peak, all while maintaining a short runtime. Consequently, four columns were tested: ACQUITY UPLC BEH C18, ACQUITY UPLC HSS T3, CORTECS UPLC T3, and YMC Triart C18 (Figure S1, Supplementary file). These columns feature various modifications of the C18 stationary phase, differing in diameter and particle size. Different solvent systems, buffers, and additives were evaluated to optimize the mobile phase composition. Acetonitrile was employed as the organic solvent, while mobile phase additives included formic acid, ammonium hydroxide, ammonium formate, ammonium acetate, and ammonium bicarbonate. Ultimately, adequate retention and a symmetrical peak shape were attained on ACQUITY UPLC BEH C18 (50 × 2.1 mm, 1.7 μm) using 10 mM ammonium formate in water as mobile phase A and acetonitrile as mobile phase B. A gradient elution program was established, featuring a total runtime of 3 min, an initial flow rate of 0.4 mL/min, and a column temperature of 40°C. Retention times were recorded at 1.36 min for both COL and COL‐d_3_. Representative chromatograms of blank human plasma and blank human plasma spiked with analytes at LOQ are illustrated in Figure 3. The chromatograms exhibit peaks without fronting or tailing, and no interference from endogenous compounds was observed at the retention times of the analytes.

In the final phase of method development, we aimed to establish a straightforward, reliable, and rapid plasma pretreatment. Consequently, the Ostro plate was selected as an alternative to techniques such as protein precipitation, liquid–liquid extraction, and solid‐phase extraction. This streamlined one‐step extraction method ensures uniform recovery, minimizes matrix effects, can be completed in just a few minutes, reduces the likelihood of errors during sample preparation (for instance, during transfers between centrifuge tubes), and facilitates high‐throughput sample pretreatment. Furthermore, as demonstrated in our earlier study, over 95% of endogenous phospholipids are eliminated during extraction (Žideková et al. 2023). This is particularly important when analyzing plasma samples using mass spectrometry with electrospray ionization, as phospholipids can significantly influence matrix effects.

To summarize, we developed a highly selective method that monitors two confirmation ions and features a robust design with a short total analysis time, requiring only 50 μL of sample. In a single‐step sample pretreatment, both proteins and phospholipids are removed. Furthermore, this approach is highly practical for conducting routine TDM in clinical laboratories.

### 3.2 Method Validation

The method was subsequently fully validated in accordance with the ICH guideline M10 on bioanalytical method validation.

The representative chromatograms of the blank plasma extract (Figure 3) did not show any significant interferences at the retention times of COL and COL‐d_3_. The interference responses were lower than 5% of the LOQ sample for the analyte and less than 0.05% of the IS.

The calibration curve, consisting of eight calibration points, was linear within the concentration range of 0.05–100 ng/mL in human plasma. For all calibrators, accuracy ranged from 95.0% to 104.8%, while precision (CV, %) was maintained between 0.3% and 5.5%. The mean of the regression equations obtained by least squares regression was *y* = 0.108805*x* + 0.000602578 (*r* ^*2*^ ≥ 0.99), where *y* represents the peak–area ratio of the analyte to the IS, and *x* denotes the concentration of the analyte. Detailed linearity validation data are provided in Table S4 (Supplementary file).

The limit of quantification was established as the lowest point of the calibration curve, at 0.05 ng/mL. The precision and accuracy of the LOQ samples were acceptable, with a CV of 3.5% and an accuracy of 107.0%. The signal‐to‐noise ratio for COL was well above 10.

By analyzing QC samples at four concentration levels, we assessed both intra‐ and interday accuracy and precision (Table 1). The intra‐ and interday precisions were below 11.9% and 7.8%, respectively. The accuracy ranged from 101.4% to 105.2%. Based on the data, we can conclude that the method demonstrates sufficient precision, accuracy, and reproducibility for quantifying COL in human plasma.

**TABLE 1:** Intra‐ and interday accuracy and precision of colchicine in human plasma.

Nominal concentration (ng/mL)	Intraday (overall mean, *n* = 6)			Interday (overall mean, *n* = 18)		
	Measured concentration (ng/mL)	Accuracy (%)	Precision (CV, %)	Measured concentration (ng/mL)	Accuracy (%)	Precision (CV, %)
0.050	0.052	103.7	11.9	0.052	103.2	7.8
0.150	0.158	105.2	4.0	0.156	104.3	2.6
40.0	41.5	103.8	2.7	40.8	101.9	5.5
80.0	83.0	103.8	0.9	81.1	101.4	3.7

The integrity of dilution was verified to analyze or reanalyze samples exceeding the ULOQ. Blank plasma samples were spiked at a concentration 10 times that of the highest calibration point. These samples were diluted tenfold with blank plasma, then processed and analyzed. The precision was found to be 1.6%, and the accuracy was 92.8%. Furthermore, the carryover test met the criterion, with no significant carryover detected in blank extracts injected after triplicate injections of the highest calibration standard. Consequently, study samples may be randomized during the analysis.

Matrix effects can lead to either signal suppression or enhancement, potentially impacting crucial method parameters, such as the limit of detection, quantification, linearity, accuracy, and precision. Therefore, these effects must be evaluated during the validation process. Matrix effects and extraction recovery were assessed at two QC levels (low and high) across six distinct blank plasma samples. The matrix effects, represented as IS‐normalized matrix factors, showed a minimal contribution of the matrix to the ionization process (Table S5, Supplementary file). The average extraction recovery for COL exceeded 80%. Although the recovery is not 100%, it remains reproducible and consistent, with a CV below 8.6% (Table S5, Supplementary file).

The stability of COL in human plasma was evaluated under various storage conditions. The results of short‐term stability, post‐processing stability, freeze–thaw stability, and long‐term stability assays at two quality control levels (low and high) are summarized in Table 2. The accuracy at each level and across all conditions remained within an acceptable range, with CVs below 2.5%. The findings indicate excellent stability of the analytes throughout the entire analytical process.

**TABLE 2:** Stability results of colchicine in plasma under different storage conditions.

Spiked concentration (ng/mL)	Laboratory, 16 h at 20°C (*n* = 6)		Autosampler, 24 h at 8°C (*n* = 6)		Three freeze–thaw cycles (*n* = 6)		Freezer, 2 months at −20°C (*n* = 6)	
	Accuracy (%)	CV (%)	Accuracy (%)	CV (%)	Accuracy (%)	CV (%)	Accuracy (%)	CV (%)
0.150	103.6	2.4	105.1	1.9	102.3	5.0	106.4	2.4
80.0	100.2	1.5	101.8	1.8	99.6	1.2	103.3	1.4

### 3.3 Method Application

The fully validated analytical method was employed to determine the plasma levels of COL in eight patient samples. The samples were collected within 5 h after the administration of COL tablets at daily doses ranging from 1 to 2 mg. The measured plasma levels varied from 0.37 to 3.15 ng/mL (median, 1.29 ng/mL). The levels of COL in plasma samples from our study are summarized in Table 3. Additionally, the therapy was deemed adequate and effective in every patient, with neither side effects nor toxic events observed.

**TABLE 3:** Characterization of patients involved in the study and measured levels of colchicine.

Patient (sex; age)	Body weight (kg)	Dose of COL per body weight (μg/kg)	Blood sampling after the morning dose	Measured plasma concentration (ng/mL)
Patient 1 (F; 61 years)	110	18	3 h	0.37
Patient 2 (F; 32 years)	60	25	3 h	1.70
Patient 3 (F; 60 years)	79	13	3 h	0.76
Patient 4 (F; 51 years)	85	12	3 h	1.28
Patient 5 (F; 32 years)	68	15	3 h	1.30
Patient 6 (M; 27 years)	80	13	1.5 h	0.95
Patient 7 (F; 47 years)	65	15	5 h	1.29
Patient 8 (F; 46 years)	55	27	3 h	3.15

*Note:* F, female; M, male.

After ingestion of standard doses, COL is quickly absorbed from the gastrointestinal tract, achieving peak plasma concentrations within 1–3 h. Peak concentrations may occur later with higher doses, suggesting a saturable influx transporter for COL in the gut. Effective steady‐state plasma concentrations are considered to be within a range of 0.5–3 ng/mL, with toxic effects occurring at approximately 3 ng/mL (Stamp et al. 2024). The biological half‐life and plasma levels may be impaired in older individuals or those with renal or hepatic dysfunction (Stamp et al. 2025). COL intoxication and side effects include abdominal pain, vomiting, diarrhea, elevated temperature, or bleeding (Stewart et al. 2020). In a study published by Canbolat (2022), plasma levels of patients with FMF taking COL in doses of 1–2 mg were found to be 1.1 ± 0.42 ng/mL. Our results are in accordance with this study, with a median plasma level of 1.29 ng/mL. However, we can observe significant variations within our study group. For example, when comparing patient No. 1 with patient No. 8, a difference of more than 10 times has been identified. Although COL is not recommended for routine TDM, there are scenarios where it may be beneficial, such as for dose adjustments (Jesenak et al. 2025).

## 4 Conclusion

In the present study, we have developed and validated a simple and sensitive LC–MS/MS method employing a high‐throughput sample preparation technique for quantifying COL in human plasma samples. Our method offers several significant advantages, including a low sample volume (50 μL) and a short run time (3 min). These factors render our method suitable for the routine monitoring of patients treated with COL. The method was successfully validated according to the ICH guideline M10 on bioanalytical method validation, which encompasses selectivity, linearity of the calibration curve, LOQ, accuracy and precision, dilution integrity, carry‐over effect, matrix effects, extraction recovery, and stability. The fully validated method was applied to the analysis of plasma samples from eight patients diagnosed with FMF and treated with COL. This could provide a tool for monitoring the appropriate concentration and its modulation by dose correction, along with assessing the achieved clinical response and safety.

## Conflicts of Interest

The authors declare no conflicts of interest.

## Supporting information


**Table S1:** Overview of selected analytical methods for the quantification of colchicine in biological matrices.
**Table S2:** Optimized multiple reaction monitoring (MRM) parameters for colchicine and internal standard (IS).
**Table S3:** Chromatographic gradient conditions.
**Table S4:** Validation data of calibration curves linearity test (*n* = 3).
**Table S5:** Matrix effects (expressed as IS‐normalized matrix factor) and extraction recovery for colchicine in human plasma samples (*n* = 6).
**Figure S1:** Comparison of different columns on the separation of colchicine.

## Data Availability

The data that support the findings of this study are available from the corresponding author upon reasonable request.
