# Supplementary material for: Development and Validation of a Rapid and Simple UHPLC–MS/MS Method for the Determination of Colchicine in Human Plasma
Source: Biomed Chromatogr. 2025 Sep 23;39(11):e70222. doi: 10.1002/bmc.70222 (PMC12457077; doi:10.1002/bmc.70222)
Supplement: Supplementary file 1 — Table S1: Overview of selected analytical methods for the quantification of colchicine in biological matrices. Table S2: Optimized multiple reaction monitoring (MRM) parameters for colchicine and internal standard (IS). Table S3: Chromatographic gradient conditions. Table S4: Validation data of calibration curves linearity test (n = 3). Table S5: Matrix effects (expressed as IS‐normalized matrix factor) and extraction recovery for colchicine in human plasma samples (n = 6). Figure S1: Comparison of different columns on the separation of colchicine. [file BMC-39-e70222-s001.docx]

**Development and validation of a rapid and simple UHPLC-MS/MS method for the determination of colchicine in human plasma**

Nela Žideková^1^, Kristián Pršo^2^, Marek Pršo^3^, Miloš Jeseňák^3,4^, Oldřich Farsa^5^, Martin Kertys^6,^**^*^**

**Affiliations:**

^1^Biomedical Center Martin, Jessenius Faculty of Medicine in Martin, Comenius University Bratislava, Martin, Slovak Republic

^2^Department of Research and Development, Saneca Pharmaceuticals, Hlohovec, Slovak Republic

^3^Department of Pediatrics and Adolescent Medicine, Jessenius Faculty of Medicine in Martin, Comenius University in Bratislava, University Hospital Martin, Slovak Republic

^4^Institute of Clinical Immunology and Medical Genetics, Jessenius Faculty of Medicine in Martin, Comenius University in Bratislava, University Hospital Martin, Slovak Republic

^5^Department of Chemical Drugs, Faculty of Pharmacy, Masaryk University, Brno, Czech Republic

^6^Department of Pharmacology, Jessenius Faculty of Medicine in Martin, Comenius University Bratislava, Martin, Slovak Republic

**Running title: LC-MS/MS analysis of colchicine in human plasma**

^*^**Corresponding author:** Martin Kertys

Department of Pharmacology

Jessenius Faculty of Medicine in Martin

Comenius University Bratislava

Malá Hora 4C

03601, Martin

Slovak Republic

Tel: +421 439279631

Email: [martin.kertys@uniba.sk](mailto:martin.kertys@uniba.sk)

ORCID iD: <https://orcid.org/0000-0002-1095-6810>

**Table S1**. Overview of selected analytical methods for the quantification of colchicine in biological matrices

| **Author (year)** | **Analytes** | **Matrix** | **Sample volume** | **Sample preparation** | **LLOQ** | **Time of analysis** | **Detection method** |
| --- | --- | --- | --- | --- | --- | --- | --- |
| Pietsch et al. (2008) | COL + 12 alkaloids | human urine and serum | 1 000 µL | SPE | 2 ng/mL serum  1 ng/mL urine | 30+ min | HPLC-UV |
| Samanidou et al. (2006) | COL | human serum/plasma, urine, blood | 100 µL | PPT/direct dilution (urine) | 1 ng/mL | 6+ min | HPLC-UV |
| Bi et al. (2022) | COL | whole blood  urine | 300 µL | LLE | 0.5 ng/mL blood  2 ng/mL urine | 3 min | LC-MS/MS |
| Bourgogne et al. (2013) | COL | human plasma | 200 µL | PPT | 0.342 ng/mL | 9.5 min | LC-MS/MS |
| Canbolat (2022) | COL | human plasma | 1 000 µL | LLE | 0.25 ng/mL | 8 min | LC-MS/MS |
| Fabresse et al. (2017) | COL + Fab bound COL | human plasma, urine and tissue | 200 µL  50–150 mg tissue | LLE | 0.5 ng/mL plasma and urine  5 pg/mg tissue | 2 min | LC-MS/MS |
| Gabani et al. (2020) | COL + febuxostat | rat plasma | 50 µL | LLE | 0.25 ng/mL | 3 min | LC-MS/MS |
| Jiang et al. (2007) | COL | human plasma | 100 µL | LLE | 0.05 ng/mL | 2.5 min | LC-MS/MS |
| Qian et al (2023) | COL | human urine and plasma | 200 µL | DSPE | 0.2 ng/mL | 8 min | LC-MS/MS |
| Shah et al. (2014) | COL | human plasma | 100 µL | SPE | 0.01 ng/mL | 1.5 min | LC-MS/MS |

COL – colchicine

DSPE – Dispersive Solid-Phase Extraction

LLE – Liquid Liquid Extraction

SPE – Solid-Phase Extraction

PPT – Protein Precipitation

LC-MS/MS – Liquid Chromatography with Tandem Mass Spectrometry Detection

HPLC-UV – High Performance Liquid Chromatography with Ultraviolet Detection

Pietsch, J., Günther, J., Henle, T., Dreßler, J., Simultaneous determination of thirteen plant alkaloids in a human specimen by SPE and HPLC. *J Sep Sci* 2008, 31, 2410–2416.

[11] Samanidou, V. F., Sarantis, G. A., Papadoyannis, I. N., Development and Validation of a Rapid HPLC Method for the Direct Determination of Colchicine in Pharmaceuticals and Biological Fluids. *J Liq Chromatogr Relat Technol* 2006, 29, 1–13.

[12] Bi, C., Gao, Y., Li, C., Gao, Y., Wang, K., He, Y., Li, Z., Yuan, H., Development, validation, and clinical application of a rapid UPLC–MS/MS method for detection of colchicine in human whole blood and urine. *Biomedical Chromatography* 2022, 36, e5437.

[13] Bourgogne, E., Soichot, M., Latour, C., Laprévote, O., Rugged and accurate quantitation of colchicine in human plasma to support colchicine poisoning monitoring by using turbulent-flow LC-MS/MS analysis. *Bioanalysis* 2013, 5, 2889–2896.

[14] Canbolat, F., Validation of Colchicine Assay Method for Therapeutic Drug Monitoring in Human Plasma. *Journal of Advanced Research in Natural and Applied Sciences* 2022, 8, 695–702.

[15] Fabresse, N., Allard, J., Sardaby, M., Thompson, A., Clutton, R. E., Eddleston, M., Alvarez, J. C., LC–MS/MS quantification of free and Fab-bound colchicine in plasma, urine and organs following colchicine administration and colchicine-specific Fab fragments treatment in Göttingen minipigs. *Journal of Chromatography B* 2017, 1060, 400–406.

[16] Gabani, B. B., Saini, N. K., Jairam, R. K., Shrinivas, P., Trivedi, R. K., Srinivas, N. R., Mullangi, R., Simultaneous determination of colchicine and febuxostat in rat plasma: Application in a rat pharmacokinetic study. *Biomedical Chromatography* 2020, 34, e4939.

[17] Jiang, Y., Wang, J., Wang, Y., Li, H., Fawcett, J. P., Gu, J., Rapid and sensitive liquid chromatography–tandem mass spectrometry method for the quantitation of colchicine in human plasma. *Journal of Chromatography B* 2007, 850, 564–568.

[18] Qian, M. rong, Chen, Z. min, Tao, X. xin, Yao, F., Xu, X. min, In-syringe dispersive solid phase filter extraction cleanup followed by liquid chromatography-triple quadrupole mass spectrometry for fast determination of colchicine in plasma/urine. *J Pharm Biomed Anal* 2023, 228, DOI: 10.1016/j.jpba.2023.115317.

[19] Shah, J., Shah, P., Patel, D., Sharma, P., Sanyal, M., Shrivaatav, P., Highly Sensitive Determination of Colchicine in Human Plasma by UPLC-MS/MS for a Clinical Study in Healthy Subjects. *Am J Mod Chromatogr* 2014, DOI: 10.7726/AJMC.2014.1006.

[10]

**Table S2** Optimised multiple reaction monitoring (MRM) parameters for colchicine and internal standard (IS)

| **Compound Name** | **MRM transitions**  **(*m/z*)** | **Cone voltage (V)** | **Collision energy (eV)** | **Dwell time (ms)** |
| --- | --- | --- | --- | --- |
| **colchicine** | 400.2 > 310.2 | 20 | 28 | 37 |
|  | 400.2 > 326.2 | 20 | 26 | 37 |
|  | 400.2 > 358.2 | 20 | 22 | 37 |
| **colchicine-d_3_** | 403.3 > 313.2 | 20 | 24 | 37 |
|  | 403.3 > 361.3 | 20 | 22 | 37 |

Underlined MRM transitions (*m/z*) are used as quantifier ions.

**Table S3** Chromatographic gradient conditions

| **Time**  **(min)** | **Flow rate (mL/min)** | **Solvent A**  **(%)** | **Solvent B (%)** |
| --- | --- | --- | --- |
| 0.00 | 0.4 | 85 | 15 |
| 0.30 | 0.4 | 85 | 15 |
| 1.40 | 0.4 | 40 | 60 |
| 1.70 | 0.6 | 5 | 95 |
| 2.20 | 0.6 | 5 | 95 |
| 2.40 | 0.4 | 85 | 15 |
| 3.00 | 0.4 | 85 | 15 |

Solvent A: 10 mM ammonium formate in water

Solvent B: acetonitrile

**Table S4** Validation data of calibration curves linearity test (n=3)

| Analyte | Nominal concentration (ng/mL) | Accuracy (RE, %) | Precision (CV, %) |
| --- | --- | --- | --- |
| **colchicine** | 0.05 | 102.0 | 3.4 |
|  | 0.10 | 95.0 | 5.5 |
|  | 0.50 | 102.5 | 1.7 |
|  | 1.0 | 104.5 | 0.3 |
|  | 5.0 | 103.2 | 1.4 |
|  | 10 | 99.8 | 0.8 |
|  | 50 | 95.9 | 1.3 |
|  | 100 | 97.1 | 0.4 |

**Table S5** Matrix effects (expressed as IS-normalised matrix factor) and extraction recovery for colchicine in human plasma samples (n=6)

| Analyte | Nominal concentration (ng/mL) | Matrix factor (CV, %) | Extraction recovery in % (CV, %) |
| --- | --- | --- | --- |
| **colchicine** | 0.15 | 0.997 (2.2) | 81.3 (7.0) |
|  | 80 | 0.986 (1.3) | 79.0 (8.6) |

The matrix effects were assessed by comparing the peak areas obtained from six individual drug-free plasma samples. The extracts of plasma samples were spiked with standard solutions of analytes at two quality control sample levels (low and high) and an internal standards solution, and compared to a pure reference standard solution in water at equivalent concentrations. The normalised IS matrix factors were calculated by dividing the analyte's matrix factor by the corresponding internal standard's matrix factor. The extraction recovery from plasma for the analyte was calculated by comparing the peak areas of quality control samples at two levels (low and high) to those of the blank sample extracts spiked with analytes at the same concentration. The extraction recoveries were investigated in plasma samples obtained from six individual sources.


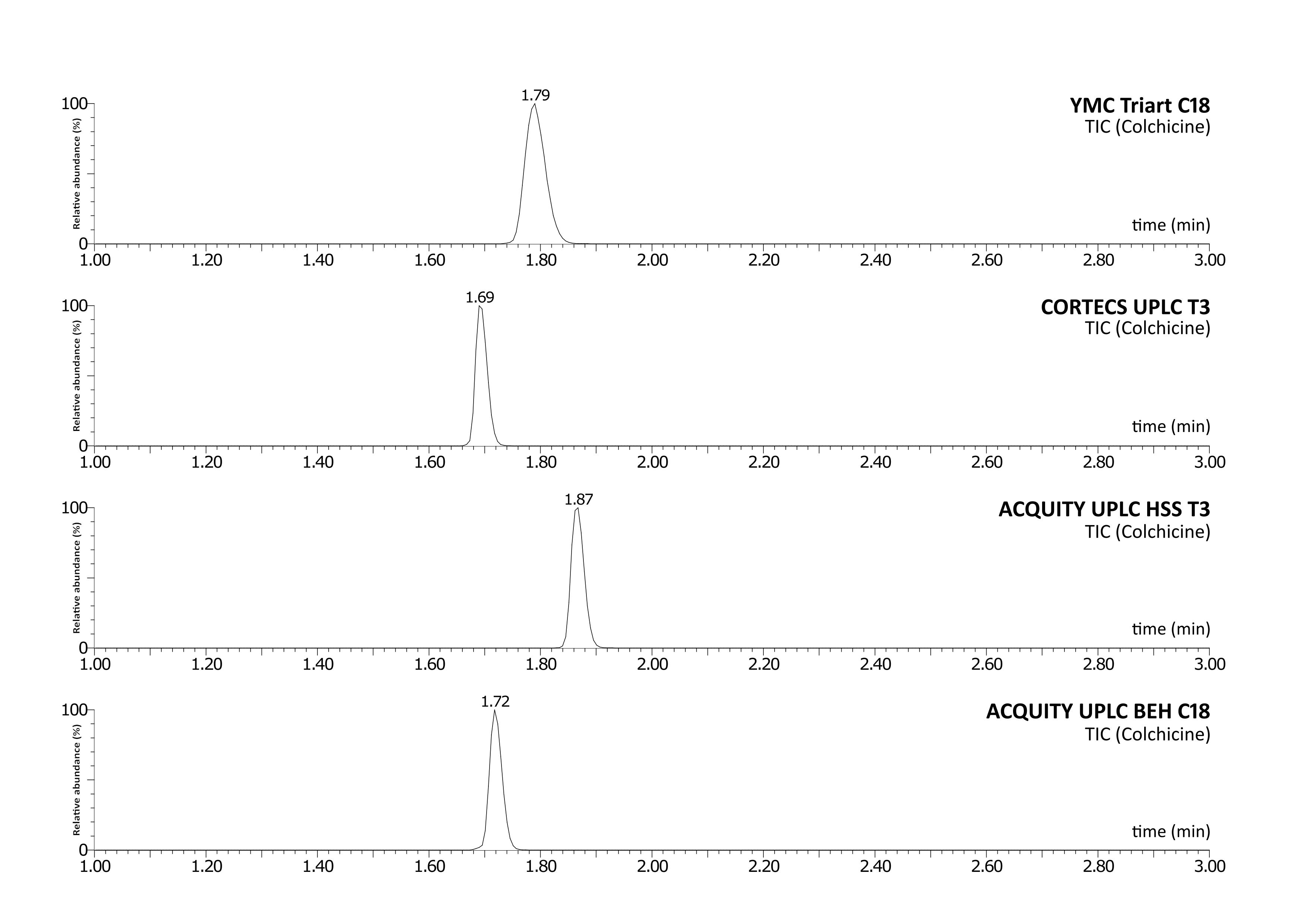


**Figure S1** Comparison of different columns on the separation of colchicine

The separation was carried out on YMC-Triart C18 (50 × 1.0 mm, 1.9 μm), CORTECS® UPLC® T3 (50 × 2.1 mm, 1.6 μm), ACQUITY UPLC® HSS T3 (50 × 2.1 mm, 1.8 μm) and ACQUITY UPLC® BEH C18 (50 × 2.1 mm, 1.7 μm).

Mobile phase A was 10 mM ammonium formate in water; mobile phase B was acetonitrile.

The elution gradient of the mobile phases was as follows: 5% to 95% B (initial, 0–4.0 min), held at 95% B (4.0–4.2 min), and then returned to 5% B (4.2–5.5 min). The flow rate was set at 0.2 mL/min for YMC Triart C18 and 0.4 mL/min for CORTECS® UPLC® T3, ACQUITY UPLC® HSS T3 and ACQUITY UPLC® BEH C18.

The temperature of all columns was maintained at 40°C. The *x*-axis represents retention time (min), and the *y*-axis represents the relative abundance (%).
